# Medical and Philosophical Intelligence

**Published:** 1814-09

**Authors:** 


### MEDICAL AND PHILOSOPHICAL INTELLIGENCE.

ROYAL SOCIETY.—On Thursday, the 30th of June, a paper by Sir EVERARD HOME, bart. was read, on the Influence of the Nerves on the beating of the Arteries. He was led to his opinion of this influence, by the case of an officer who had received a ball in the leg. The ball was lodged among the fractured parts of the tibia; and after its extraction, an attempt was made to remove some parts by the application of caustic alkali; but the pain produced was so great that they were obliged to desist. The pain was not in the part to which the alkali was applied, but at some distance, and seemed to result from the violent beating of the arteries. Hence it was ascribed to the action of the alkali on a nerve, and the consequent reaction of this nerve upon the arteries. Upon laying bare the carotid artery of a rabbit, and applying caustic alkali to the intercostal nerve, the artery began to beat violently, and continued to do so for some time. This fact, in the author's opinion, throws considerable light on the action of the arteries in various parts of the animal economy, hitherto but imperfectly explained.

---

M. BERTRAND has recently made some experiments, which lead us to hope that charcoal will be found a useful antidote in cases of mineral poison taken into the stomach.

*Super-oxymuriate of Mercury.*—Exp. 1. On 2d of February, 1811, at ten o'clock in the forenoon, I caused a dog, six months old, to swallow, on an empty stomach, six grains of corrosive sublimate and eight grains of charcoal mixed together, and enclosed in a portion of the intestine of a fowl, tied at its two extremities. The animal suffered no inconvenience from it. He ate his food with good appetite in the evening, as well as on the following days.

Exp. 2. On the 24th of the same month, at ten minutes past ten o'clock in the morning, the same dog swallowed six grains of super-oxymuriate of mercury, mixed with some butter. In a quarter of an hour he fell into violent struggles, and soon after vomited some glairy fluid mixed with blood. He appeared to be in great pain, and hung down his head, sometimes lying on the ground; his jaws were closed by tetanic spasm. At twenty minutes past one, I caused him to swallow some water with charcoal, insinuating it at the corners of his mouth. The efforts at vomiting became less violent and less frequent. At forty minutes past one I gave him another dose of the charcoal powder, which this time was rendered thicker, as the animal could swallow better, the jaws not being so firmly closed, and the vomiting had entirely ceased. At half past two the dog still appeared dull, but tranquil; he refused food, and prevented other dogs from approaching it by the most furious attacks. At five o'clock he appeared to suffer some tenesmus, and begun to take a little food. The next day all the functions were performed in a healthy manner.

Exp. 3. On the 6th of February, 1813, at eight o'clock in the morning, morning, I swallowed, fasting, four grains of corrosive sublimate in a cup of strong mixture of charcoal powder, sweetened, and rendered aromatic by orange-flower water. In twenty minutes I perceived an oppressive pain in the præcordial region, with some heat in the stomach. I felt a slight sensation of thirst during an hour, but took nothing to allay it; at ten o'clock, not feeling the least degree of pain, I breakfasted with an appetite, and experienced no further inconvenience.

*Arsenious Acid.*—Exp. 1. On the 2d of February, 1811, at five minutes before ten o'clock in the forenoon, I gave to a dog, seven months old, on an empty stomach, six grains of powdered arsenic, mixed with eight grains of charcoal, enclosed in a piece of the intestine of a fowl. He appeared to suffer no inconvenience from swallowing it; he continued perfectly well, and enjoyed his usual appetite; three days afterwards he voided the portion of intestine completely empty.

Exp. 2. On the 24th of February, 1811, at noon, I caused a dog, nine months old, to swallow, on an empty stomach, six grains of arsenic mixed with butter. In half an hour he vomited, with violent efforts, mucus slightly tinged with blood. Some charcoal mixture was given to him at a quarter before one, and all the symptoms were speedily alleviated. At two o'clock he swallowed another dose of the decoction; at half past two all the functions seemed perfectly recovered, and he had a good appetite; at five o'clock he ate heartily, and with voraciousness.

Exp. 3. At half past seven o'clock in the morning of the 16th of February, 1813, I swallowed fasting five grains of arsenic powder in half a glass-full of a strong mixture of charcoal. At a quarter before eight o'clock, I perceived a painful sensation of heat in the epigastric region, with great thirst, but without any other symptom of consequence. I now drank another half glass of the mixture. At half past nine the oppressive pain in the epigastrium had disappeared, but seemed to have changed its situation, as I felt a similar sensation in the rest of the alimentary canal. Being very thirsty, I drank several cups of infusion of orange flowers; and at a quarter past ten I was completely well. At noon I dined as usual, without inconvenience, and could perceive no farther derangement in any of the digestive functions.

---

*Substance extracted from the Vagina of an old Woman.*—Dr. Thomson has received from a surgical friend a specimen of a substance which had been extracted from the vagina of an old woman. It is of a yellowish white colour, smooth upon the outer surface, has the hardness of bone, and easily breaks. When heated it gives out the smell of burning feathers, and becomes black; but if it be kept for some time red-hot, it becomes quite white, and has the aspect of burnt bone. It dissolves slowly, and without effervescence in muriatic acid, from which it may be again precipitated by caustic ammonia. These properties leave no doubt that it consists of phosphate of lime, cemented together by an animal substance which is probably probably of the nature of mucus. When a portion of this matter is kept for some days in a weak acid, almost the whole dissolves, and the undissolved portion is in a state of white flocks.

---

*Report of the State of Vaccination in Sweden.*—On the 14th of January, 1814, Mr. Macmichael, an English gentleman, attended the Royal College of Health in Stockholm, and delivered to the college a copy of the Report of the National Vaccine Establishment in London, dated the 22d April, 1813, and presented to Lord Sidmouth, Secretary of State for the Home Department; at the same time he requested, that a short account of the progress of vaccination in Sweden, and of the measures which had been adopted for its promotion, might be communicated to him, for the information of the British Parliament.

The Royal College had particular satisfaction in receiving Mr. Macmichael, and undertook to comply with his request so much the more readily, as it had the pleasure of numbering among its honorary members the respectable name of Dr. Jenner for whom it was reserved to demonstrate, by the most decisive experiments, the protective power of the cow-pox against the most terrible and destructive contagion of the small-pox; a pestilence, which, by means of this blessed discovery, must certainly be ultimately extirpated from the face of the earth.

It was to be expected, from the excellent arrangements which the Kings of Sweden had adopted for somewhat more than half a century, in every department of medical science, that the incomparable discovery of Dr. Jenner, like the inoculation for the small-pox at a former period*, should not only become an object of the most accurate investigation, but also, when approved by experience, be generally introduced and promoted by rewards and established regulations.

The medical practitioners of Sweden, who had already been informed, from the time of Dr. Jenner's first discovery, by means of a constant correspondence with the learned in other countries, of the expectations which were entertained of the success of experiments made with the cow-pox, had great pleasure in learning that one of their colleagues, Dr. Gahn, a member of the Royal College, had, towards the end of 1799, procured some vaccine matter, and obtained the most satisfactory result from inoculating with it. Another Swedish physician, now Professor of Medicine, Dr. Rosensköld, printed in 1801 a small pamphlet, entitled, "to the Pub-

---

* It is remarkable, that the celebrated Dr. David Schultzenheim, who was appointed as long ago as 1754, by the States of the kingdom, to inquire into Sutton's and Dimsdale's mode of inoculating the small-pox in England, is now the president of the Royal College of Health, and has been the most instrumental, by means of his powerful influence, in promoting the most salutary measures for the introduction of vaccination.

tise on the Cow Pox;" and performed vaccination with success in several parishes in Skaine. About the same time the undersigned also published a more detailed account, with coloured figures, under the title, "the Small Pox may be extirpated;" and this essay was distributed to all the churches in the kingdom.

The government, already attentive to the inestimable advantage which the inoculation of the cow-pox seemed to promise, directed the college to examine Dr. Jenner's discovery with the greatest accuracy, for which the proper means were immediately afforded; and the College was ordered, after collecting the results, to present its report to the King.

This report, which fully confirmed the excellence of the Jennerian discovery, occasioned the salutary law which was first enacted in 1803, by which vaccination was established throughout the kingdom; and the College was commanded to promote its adoption by all possible means. The king was pleased to appropriate 900 dollars, spec. banco, to be divided into premiums, which were to be distributed among such medical men as could exhibit the greatest number of vaccinated persons.

A particular regulation was made for the metropolis, imposing a fine of three dollars on any one who should fail to announce to the Medical Officer of the district, the appearance of the contagion of the small-pox; and in every such case, the persons infected was to be carried to the Small Pox Hospital, where every measure was adopted for his being properly nursed; and the same precautions have been continued to the present time.

It was long a question, whether new-born children could be vaccinated with success, and whether the matter taken from them might be employed with as much security as if taken from adults?

This doubt has been altogether removed, and in the General Lying-in Hospital all the children are now vaccinated within nine days from the time of their birth; so that, by means of this progressive vaccination, fresh matter remains constantly in existence.

The want of a sufficient supply of vaccine matter for the extensive provinces of the kingdom, was long an obstacle to the universality of vaccination in Sweden. This obstacle no longer exists; since the Royal College of Health, in consequence of the humble representations which it made to the King, obtained, the adoption of a very effectual measure for this purpose, in the appointment of a particular establishment for the general regulation of vaccination throughout the kingdom, which took place in the year 1812.

This establishment consists of a director, and several inspectors of the stations for vaccination in the provinces. The director is a member of the Royal College of Health, whom the King has graciously commanded to receive and examine all reports, to answer all inquiries, to conduct the distribution of vaccine matter, which is delivered, free of postage, to all persons who apply for it; and lastly to report to the College every thing relating to vaccination that requires further regulation, and to propose to it as proper persons to receive rewards, all those who appear to be the most deserving. He has also the immediate inspection of all the medical men, who are appointed to conduct the business of the stations, established in almost every province; the progressive vaccination performed at these stations being calculated to maintain a constant supply of fresh matter, which is also distributed, free of postage, to those who require it; and their proceedings being registered in proper catalogues and journals.

In Stockholm, three several stations of this kind have been appointed, whence fresh matter may always be procured with certainty, if it happens to be wanting in any particular province.

The archbishop, bishops, and the whole of the clergy throughout the kingdom, having, from the time of the happy discovery of vaccination, embraced it with the utmost distinguished zeal; and many of this respectable body having not only employed the most effectual means for the removal of vulgar prejudices against it, but having even actually practised vaccination themselves; the King, assured of the continued exertions of the clergy in the same cause was pleased to direct, that every minister should superintend the progress of vaccination within his parish; and should be empowered to call to his assistance one or more inspectors of vaccination, according to circumstances, for the purpose of causing all children to be properly vaccinated within the first year after their birth, and keeping proper documents of the performance of the operation. In each parish or district there must be an accredited vaccinator, whose duty is to perform vaccination, and to give a report of his proceedings to the Royal College of Health.

The College has also published, by the King's command, a book of instructions for vaccinators and inspectors of vaccination, which has been distributed gratis to all the Churches in the kingdom. This treatise adapted to the use of the Public, affords an accurate knowledge of the true and false cow-pox; of the varieties which most frequently occur in it; and of the cutaneous diseases which occur so often in Sweden, very nearly resembling the small-pox.

For the more effectual encouragement of the practice of vaccination, the King has been graciously pleased to appoint rewards of two different kinds, pecuniary premiums and honorary medals. The latter are distributed, commonly in silver, but sometimes in gold, to those who have particularly distinguished themselves. In all cases those who have deserved rewards, are humbly pointed out to the King, by the College of Health; and his majesty has reserved to himself the right of assigning the proportions in which those rewards shall be distributed. It is also in the King's name, and with a certain degree of publicity, that these marks of his approbation are bestowed.

For the honour of the medical profession in Sweden, it must not be forgotten, that although inoculation for the small-pox was one of the most lucrative branches of their private practice, and has been entirely superseded by the simple process of vaccination, no one individual of the profession has raised any obstacles against the cowcow-pox; but every one has contributed to its advancement, by giving advice, information, and assistance, to the utmost of his ability. No single publication has appeared to call in question its high importance*, and its superiority to variolous inoculation; which has been entirely discontinued ever since the year 1802, rather by a tacit and universal consent, than in consequence of any royal prohibition.

It may therefore be asserted, that the small-pox, that equally disgusting and destructive pestilence, which for many ages continued annually to send out of the world an immense number of young children, is now through the influence of Dr. Jenner's inestimable discovery, so perfectly extirpated in Sweden, that it never can become epidemic, even if at any time notwithstanding all the orders and all the vigilance employed for its exclusion, the infection should make its appearance. Such in the last twelve years, has been the effect of the King's wise and humane attention, of the unanimity and disinterestedness of the medical profession, of the patriotic zeal of the clergy, of the good examples so promptly exhibited by the upper classes, and of the progress of information and civilisation in the lower.

The undersigned, who has drawn up this short account at the request of the Royal College of Health, has also the honour of sending with it, in the name of the College, a copy of the book of instructions, and an impression in silver of the honorary medal, which was struck by the King's command, under the direction of the College, and which is distributed in the King's name, for the promotion of vaccination.

FR. HEDIN, M. D. First Physician to the King,
Medical Councellor, &c. &c.

*Stockholm*, 10*th February*, 1814.

---

*On the Solubility of White Oxide of Arsenic in Water;* by M. KLAPROTH.—The solubility of white oxide of arsenic in water is a property which essentially characterizes it. Though the fact has been long known, yet the degree of solubility has not been accurately determined. According to Bergman, eighty parts of water at the temperature of 60° dissolve one part of white oxide of arsenic, while the same quantity of oxide is dissolved by fifteen parts of boiling water; according to Navier, eighty parts of boiling water are requisite to dissolve one of the oxide; and according to Hagen,

---

* The answer which the undersigned returned the 1st Nov. 1801, to a letter addressed to him, by the Vaccine Committee of the Society of Medicine at Paris, and which is inserted in the second Report of that committee, cannot justly be considered as a publication of this kind. It was not quite three months after this time, that having acquired perfect confidence from inoculating a cow, with the cow-pox, and transferring the operation to the human subject, he published the before-mentioned essay entitled, "the Small-Pox may be extirpated."

 30 grains

30 grains of white oxide of arsenic require four ounces of boiling water to dissolve them.

These different statements induced me to endeavour to ascertain the proportion; and the result of my experiments is, that three parts of white oxide of arsenic may be kept in solution by 100 parts of water at a medium temperature. On that account, the statement of Aschof, that one part of white arsenic requires 200 parts of boiling water to dissolve it, appeared to me extraordinary.

As this result may occasion mistakes in medical jurisprudence, I consider it as proper to point out the error of M. Aschof, and to show that the new experiments which I have repeated on this subject have confirmed my former opinion.

A.—To determine the solubility of the oxide in cold water, I introduced 20 grains of it, previously reduced to a fine powder, into a flask containing 10 ounces of water of the temperature of 60°. This mixture was left for 24 hours, being often agitated in the mean time. The undissolved portion, collected upon a filter, and well dried, was eight grains. Of consequence, 12 grains had been dissolved. The result of this experiment is, that 1000 parts of cold water dissolve only 2½ parts of white oxide of arsenic.

B.—Water can only saturate itself with this oxide at the boiling temperature. To ascertain the degree of solubility, I boiled for a quarter of an hour 200 grains of white oxide of arsenic in powder in four ounces of water in a phial. As soon as the undissolved part was deposited, I decanted off the liquid portion, which weighed 1800 grains. This liquid, evaporated in a capsule, the weight of which had been determined beforehand, left for residue 140 grains of white oxide of arsenic. Therefore 1000 parts of boiling water take up 77¾ of white arsenic.

C.—It was particularly important to know how much white oxide of arsenic the boiling water would retain after it was cold. For this purpose 10 ounces of boiling water were saturated with white oxide of arsenic in powder. After cooling I left the phial for three days in cold water, during which time some white arsenic separated in the crystalline form. Five ounces of the decanted solution were evaporated in a capsule previously weighed. The residue, when well dried, weighed 72 grains, and was white oxyd of arsenic. Hence it follows that 1000 parts of water retain in solution, after cooling 30 parts of white oxyd of arsenic, or 100 parts of water retain three parts of the oxide. It is obvious that the cold of winter may produce some modification in these proportions.

D.—The crystalline form of the white oxide of arsenic obtained by evaporation may lead to the suspicion of the presence of water, or that the oxide is in a state of an hydrate, which might account for the argumentation of its weight. To determine this point, I boiled three ounces of water in a phial with 100 grains of arsenic. After a quarter of an hour's boiling, all the arsenic was dissolved. The clear solution, being evaporated to dryness, left 100 grains of white oxyd of arsenic in a crystalline form. This experiment shows that oxide of arsenic does not combine with water when dissolved in that liquid, and evaporated to dryness.—*Thompson's Annals of Philosophy.*

We select the following paragraph from the Morning Chronicle, dated August 2d, giving the account of the use of Clivers, or Goose-grass, as a remedy for *cancer*. We do not vouch for the truth of the statement, which it would be equally arrogant in us to deny; nor shall we question whether the disease cured was actual cancer, but simply remark, that if it were not so, admitting the fact, it may be received as an evidence at least that the medicine is serviceable in *some* ulcerations—*mali moris.* The cause of science would be much advanced if our expectations from new remedies were more moderate. The process was recommended by the minister of a parish in the country to a poor woman, who had been for many years afflicted with a bloody cancer, and who was then thought to be in so hopeless a state as to have but a short time to live. After giving her an aperient medicine, advising her to abstain from salt meats, and to live on the most simple diet, he advised her to take, twice a-day, a quarter of a pint of the juice of clivers, the plant having been well pounded and squeezed; he ordered that the juice should also be boiled, and mixed with hog's lard, for an ointment to the wound, laying the bruised clivers over it, and keeping them constantly applied and renewed. The amendment to be expected is so very gradual, that it requires steady perseverance in the use of both the internal and external means. In three months the poor woman was cured, and the wounds perfectly healed; and she now repeats the regimen every spring and fall, for prevention. The same benevolent clergyman recommended the process to a gentleman who had a troublesome eruption, somewhat like a leprosy; and he, in addition to the rest, mixed clivers with his salad. In a few months he was perfectly well. It was also given to a poor man in Herefordshire, who had a cancer in his face to a dreadful degree, and he was perfectly restored by it. It is also said to be frequently beneficial in consumptive cases, as well as in other scorbutic complaints. Encouraged by the account of the benefit derived from the use of this plant, a lady was induced, last January, to send the particulars here related to a person in Kent, who, she understood, was labouring under that sad disease, and suffering exquisite pain from it. She has persevered in the remedy, without intermission, for three months; and writes word now, that she hopes, by the Divine Blessing, to be entirely cured of a disorder which had afflicted her fourteen years. The tumours are healed, except one, which is reduced to the size of a pin's head: she feels no pain, and says that her health and spirits are excellent.

---

*Report of the Diseases in Paris for June and July.* By MM. Menuret, Portal, Bazin, Duffour, De Montegre.—The weather at Paris during the last fortnight in June, was extremely cold. The effects of the temperature were manifested by the continuance, the number, and the obstinacy of catarrhal and rheumatic affections; and the cold was more severely felt, and proved more injurious, from its suddenly occurring after very hot weather. Inflammatory complaints were not frequent; humoral, bilious bilious fevers were more common. Stomach affections, and diarrhœas, which, from its scarcity, could not be attributed to the abuse of fruit, nor, from the cold state of the season, to the warmth of the weather, were very prevalent; they were more probably caused by checked perspiration, and readily yielded to ipecacuanha and tonics.

Several eruptive disorders were noticed; amongst these were cases of *scarlatina, rubeola,* and (with regret we mention it) *variola,* a melancholy proof of invincible obstinacy and prejudice.

Typhus fevers have diminished in number and intensity, but it still exists amongst some of the foreigners lately arrived at Paris; and there is reason to suppose that they had occupied the same lodgings if not beds that had been recently occupied by persons suffering under that species of fever.

The beginning of July the heat became suddenly augmented, Reaumur's thermometer rising to 18° and 20° in the middle of the day. For some days an universal calm prevailed. On the evening of the 7th instant a storm threatened, but passed away, and on the following day the heat was increased. This state of the atmosphere was almost insupportable; not a breath of air was perceptible whilst the sun was above the horizon. The immediate effects are characterized by numerous putrid fevers; almost all diseases have now a tendency to put on the type of these fevers. Cutaneous eruptions, and cases of obstinate ophthalmia, are also very prevalent and obstinate. The authors of the report condemn the use of purgatives in the treatment of these putrid fevers, as they term them, and seem very fearful of lowering their patient; but they do not recommend any particular plan, nor acquaint us with the success of any remedies that may have been employed. They quote Hippocrates against the use of purgatives in the commencement of diseases; and gravely remark themselves, "On ne doit pas oublier que presque j'amais il n'est avantageux qu'il y ait des évacuations alvines dans le commencement des maladies." The difference of practice, in this respect, between the two countries, is very striking.

---

The Bulletin of the *Societé Medicale d'Emulation* contains two authenticated cases of measles occurring twice in the same individual—one of a lady who was attended by M. Gaslelie in 1781, and again in 1813, which is said to have possessed all the characters of authenticity that could be desired. At the same time M. Montaign, Physician of the Hotel Dieu, had a patient with the measles who before had gone through the disease.

---

*Hooping Cough.*—M. Bertrand, having vaccinated twenty-one children labouring under the hooping cough, draws the following conclusions. 1. That the progress of vaccination, even in its most perfect state, produces little or no effect at the attack or during the first stage of hooping cough. 2. That the cough is alleviated by vaccination, when the disease has arrived at its second stage. 3. That, when the hooping cough is in its last stage, vaccination produces a remarkable and, as it were, a specific effect upon it. In the the first stage it produced only a slight effect upon one out of nine children. In the second stage the disease was sensibly influenced in five out of seven children; and in the third stage, out of five children, one obtained an immediate cessation of the cough, and three were sensibly relieved during its further progress. On one it produced no effect; but the strength of this child was so reduced, that the vaccine pustule, although possessing its true character, went through its course but very imperfectly.

---

M. de MONTEGRE has made several observations relating to digestion, which have led to the following inferences:—1st, That the gastric juice, when it is not acid, putrefies like saliva. 2d, That it never exercises an antiseptic power on the food but when it is acid; and that saliva which has acquired an equal degree of acidity by means of acetic acid, produces the same effect. To discover whether acidity was a condition indispensible to digestion, M. Montegre gave a dose of magnesia before eating, which was suffered to saturate the whole of the acid contained in the stomach. The food, after a certain time, being vomited, it was found that digestion had begun, but there was no appearance of acidity. When it was vomited still later, it was much more digested and manifestly acid.

The author draws these conclusions from his observations:—The gastric juice does not appear to differ from saliva, and is merely a solvent proper to prevent the putrefaction of the aliment, and to promote their digestion, independant of the action of the stomach. The acidity which it possesses, as also that which the food receives, ought to be attributed to the action of the stomach.

---

Dr. HARRISON, Lecturer on Medicine at Windmill-street, has been elected one of the Physicians to the Northern Dispensary, in the place of Dr. WHITTELL, resigned.—Mr. ANDREW MATHIAS, of Bloomsbury-place, has been elected Consulting Surgeon to the same Institution, in the place of Mr. CHARLES BELL, resigned, and now one of the Surgeons to the Middlesex Hospital.

---

About a fortnight since died, at a very advanced age, Dr. BENAMOR, of Millman street, Bedford-row, whom we believe, of late years, to have been the only practiser of animal magnetism of this country.

---

Dr. TROTTER, of Newcastle, is preparing for the press a new work, entitled, Reflections on the Diseases of the Poor for the last ten Years; being a summary of those patient's cases who have received his gratuitous advice on the mornings of Tuesday and Saturday, in number upwards of 3000. Dr. Trotter has taken for his model the fine disquisitions of Dr. Reid, which were published in the Monthly Magazine a few years ago, and will comprehend many of the complaints incident to the laboring poor, in the mining, manufacturing, and other departments of the neighbourhood.

 The

The Medical Lectures in the University of Glasgow will begin on Tuesday, Nov. 1st, at the following hours:—Institutions of Medicine, by Dr. FREER, at half-past eight in the morning. Surgery, by Dr. JEFFRAY, at ten. Midwifery, by Mr. TOWERS, at eleven. Practice of Medicine, by Dr. FREER, at twelve. Anatomy, by Dr. JEFFRAY, at two in the afternoon. Dietetics, Materia Medica, and Pharmacy, by Dr. MILLAR, at three. Chemistry and Chemical Pharmacy, by Dr. CLEGHORN, at seven.

The following gentlemen have obtained the degree of Doctor in Medicine, from this University, within the last twelve months:—Mr. B. Blake; Mr. Shirley Palmer; Mr. Charles Scudamore; Mr. Joseph Moore; from England. Mr. William Hunter; Mr. James Wright; Mr. William Cumin; from Scotland. Mr. Llewelyn Jones; from Wales. Mr. John Kennedy; Mr. James Turretin; Mr. James Kinneir; from Ireland.

---

Dr. BROWN will commence his Lectures on Botany about the beginning of May next.

---

Mr. WANT will deliver a Course of Lectures on the Theory and Practice of Midwifery, early in the month of October.

---

Dr. ADAMS will commence his autumnal Course of Lectures on the Institutes and Practice of Medicine, at his house, No. 17, Hatton Garden, on Tuesday the 4th of October, at ten o'clock in the morning precisely.

---

*St. Thomas's and Guy's Hospitals.*—The winter Course of Lectures at these contiguous Hospitals will commence in the first week of October: viz.

At St. Thomas's.—Anatomy, and the Operations of Surgery, by Mr. ASTLEY COOPER and Mr. HENRY CLINE. Principles and Practice of Surgery, by Mr. ASTLEY COOPER.

At Guy's Hospital.—Practice of Medicine, by Dr. BABINGTON and Dr. CURRY. Chemistry, by Dr. BABINGTON, Dr. MARCET, and Mr. ALLEN. Experimental Philosophy, by Mr. ALLEN. Theory of Medicine, and Materia Medica, by Dr. CURRY and Dr. CHOLMELEY. Midwifery, and Diseases of Women and Children, by Dr. HAIGHTON. Physiology, or Laws of the Animal Economy, by Dr. HAIGHTON. Structure and Diseases of the Teeth, by Mr. FOX.

These several Lectures are so arranged, that no two of them interfere in the hours of attendance; and the whole is calculated to form a complete course of medical and chirurgical instruction.

---

*Theatre of Anatomy, Bartlett's Court, Holborn.*—Lectures on Anatomy, Physiology, Pathology, and Surgery, by Mr. JOHN TAUNTON, F.A.S. Member of the Royal College of Surgeons of London, Surgeon to the City and Finsbury Dispensaries, City of London Truss Society, &c. will commence on Saturday, October 8, at eight o'clock in the evening precisely, and be continued every Tuesday, Thursday, and Saturday, at the same hour.

Dr. RAMSBOTHAM will begin his winter Course of Lectures on the Science and Practice of Midwifery, including the Diseases of Women and Children, on Thursday, October 6th, at his house, No. 9, Old Jewry.

---

*Theatre of Anatomy, Blenheim-street, Great Marlborough-street.*—The autumnal Course of Lectures on Anatomy, Physiology, and Surgery, will be commenced on Saturday, the 1st of October, at two o'clock, by Mr. BROOKES.

Anatomical Converzationes will be held weekly, when the different subjects treated of will be discussed familiarly, and the student's views forwarded. To these none but pupils can be admitted.

Spacious apartments, thoroughly ventilated, and replete with every convenience, are open all the morning, for the purposes of dissecting and injecting, where Mr. Brookes attends to direct the students, and demonstrate the various parts as they appear on dissection.

---

Dr. PEARSON, having delivered distinct Courses of Lectures, during twenty-six years, on the Theory and Practice of Physic, on Chemistry; on the Materia Medica, with Medical Botany, purposes in future to confine himself to the subject of the Laws of the Animal Œconomy and the Practice of Physic. His Course will commence the first week of October, as usual, in George-street, Hanover-square, from nine till ten every morning. The Lectures in the other departments, viz. on the Materia Medica, on Demonstrative Pathology, with Clinical Lectures on Medical Jurisprudence, will be delivered by Dr. ROGET and Dr. R. HARRISON; and a full Course in Chemistry will be given by Dr. JOHN DAVY, at the Theatre in Windmill-street.

---

Dr. CLARKE and Mr. CLARKE will begin their winter Course of Lectures on Midwifery and the Diseases of Women and Children, on Tuesday, October 4th. The lectures are read every morning from a quarter past ten to a quarter past eleven, at Mr. Clarke's house, No. 11, Saville-row, for the convenience of students attending the Hospitals.

---

Dr. CLOUGH commences his autumnal Course of Lectures on the Science and Practice of Midwifery, including the Diseases of Infants, on Monday the 10th of October.

---

Dr. MERRIMAN will commence his usual Course of Lectures at the Middlesex Hospital, on Monday, October 3d, at half-past ten o'clock. These Lectures embrace a general view of the Theory and Practice of Midwifery, and the Diseases of Women and Infants.

---

Dr. CLUTTERBUCK will begin his autumnal Course of Lectures on the Theory and Practice of Physic, Materia Medica, and Chemistry, on Wednesday, October the 5th, at ten o'clock in the morning, at his house, No. 1, in the Crescent, New-Bridge-street, Blackfriars.

---

*Anatomical Theatre, Lower College-street, Bristol.*—Mr. THOMAS SHUTE will commence his winter Course of Lectures on Anatomy, Physiology, and Surgery, on Saturday, October the 1st, at eight o'clock in the morning.

